# Quality of referrals for elective surgery at a tertiary care hospital in a developing country: an opportunity for improving timely access to and cost-effectiveness of surgical care

**DOI:** 10.1016/j.ijsu.2015.01.033

**Published:** 2015-02-04

**Authors:** Adam Gyedu, Emmanuel Gyasi Baah, Godfred Boakye, Michael Ohene-Yeboah, Easmon Otupiri, Barclay T Stewart

**Affiliations:** Department of Surgery, School of Medical Sciences, Kwame Nkrumah University of Science and Technology, Kumasi, Ghana; Komfo Anokye Teaching Hospital, Kumasi, Ghana; Department of Surgery, Komfo Anokye Teaching Hospital, Kumasi, Ghana; Department of Surgery, Komfo Anokye Teaching Hospital, Kumasi, Ghana; University of Ghana Medical School, Accra, Ghana; School of Public Health, Kwame Nkrumah University of Science and Technology, Kumasi, Ghana; Department of Surgery, University of Washington, Seattle, WA, USA

**Keywords:** referral, surgery, health systems, developing country

## Abstract

**Introduction:**

A disproportionate number of surgeries in low- and middle-income countries (LMICs) are performed in tertiary facilities. The referral process may be an under-recognized barrier to timely and cost-effective surgical care. This study aimed to assess the quality of referrals for surgery to a tertiary hospital in Ghana and identify ways to improve access to timely care.

**Methods:**

All elective surgical referrals to Komfo Anokye Teaching Hospital for two consecutive months were assessed. Seven essential items in a referral were recorded as present or absent. The proportion of missing information was described and evaluated between facility, referring clinician type and whether or not a structured form was used.

**Results:**

Of the 643 referrals assessed, none recorded all essential items. The median number of missing items was 4 (range 1 – 7). Clinicians that did not use a form missed 5 or more essential items 50% of the time, compared with 8% when a structured form was used (*p*=0.001). However, even with the use of a structured form, 1 or 2 items were not recorded for 10% of referrals and up to 3 items for 45% of referrals.

**Conclusion:**

Structured forms reduce missing essential information on referrals for surgery. However, proposing that a structured form be used is not enough to ensure consistent communication of essential items. Referred patients may benefit from referrer feedback mechanisms or electronic referral systems. Though often not considered among interventions to improve surgical capacity in LMICs, referral process improvements may improve access to timely surgical care.

## Introduction

For many reasons, health systems in low- and middle-income countries (LMICs) struggle to deliver optimum care. Specialties, such as surgical services, record low coverage rates despite being an essential component of public health and quality healthcare systems.[1-3] As a result, unmet surgical needs in low- and middle-income countries (LMICs) are both large and increasing.[4] Despite more than 96 million operations performed annually, community-based surveys of unmet surgical needs from developing countries have demonstrated that 5 - 10% of individuals live in need of at least a surgical consultation, and often an operation.[3, 5-9] Further, 20 - 35% of people who die in LMICs could have either been cured, treated or palliated by timely surgical care.[10] This gap is the result of dire insufficiencies in surgical infrastructure, equipment and personnel and is common to all LMICs.[11-17] Given these deficiencies, operations are concentrated in secondary and tertiary facilities and in urban centers where necessary inputs are more readily available.[18]

The time between a person developing surgical need, presenting to a local health facility, being assessed and investigated to obtaining a working diagnosis is prolonged in most LMICs for several reasons.[19] Consequently, patients routinely present in late stages of their disease and do often not receive the full benefits of surgical care.[20, 21] Reasons for this include lack of access to care, unaffordable direct and indirect costs of care, and waiting times between referring and receiving hospitals.[22, 23] While overcoming system-wide access to and cost of care barriers require significant investment and political will, referral processes can be improved with simple, cost-effective interventions.[22] Several successful process improvements from high-income countries (HICs) have been described, such as the use of structured referral letters, the same have not been reported from LMICs.[22, 24] Understanding the potential benefits of standardizing the referral process, the Ghana Health Service (GHS) employed a structured referral form; however, it has not been widely adopted outside of government facilities.[25]

In order to determine whether there was an opportunity to strengthen surgical service delivery by standardizing the referral process, a quantitative assessment of the quality of referrals for surgery to a tertiary hospital in Ghana was performed. By doing so systematically, uniform deficiencies in requisite information for timely surgical care could be identified and a targeted intervention developed.

## Materials and methods

### Ethics

Collection of de-identified data routinely used to sort referrals for consultation and used for quality improvement purposes met criteria for exemption of ethical review by Komfo Anokye Teaching Hospital and the University of Washington ethical review committees.

### Data collection and analysis

All referrals for elective surgical evaluation to Komfo Anokye Teaching Hospital in Kumasi, Ghana between August and October of 2013 were assessed for presence or absence of essential items. Essential items were defined as: those reported by published consensus guidelines; items on the GHS structured referral form; or information that would decrease wasteful duplication of limited diagnostic resources (i.e. diagnostic tests and treatments to date).[26-32] These items were:

1. Patient's age;
2. Working diagnosis;
3. Reason for referral;
4. Abbreviated history of illness;
5. Whether or not laboratory tests or diagnostic imaging was performed (or results if performed);
6. Medical history or treatment provided to date; and
7. Surgical history or treatment provided to date.

Other items, such as contact information of referrer or patient, referring facility or language needed for effective communication are desirable, they were not considered essential on referral forms (Table 1). Assessment criteria were designed to be inclusive. Specifically, an item was considered absent only if none of the following were documented: i) the item having been asked or performed; ii) the item *not* having been asked or performed; or iii) the result of the item (e.g. patient information, medical/surgical history or result of diagnostic test) if asked or performed. Lastly, note was taken if referrals were done without a form, using a non-GHS structured form or using the GHS structured form.

Two physicians assessed referrals (AG and EGB) and a third was available in case of discrepancies (MOY). Data were entered into Excel (Microsoft, Redmond, WA, USA) and described with Stata v13 (College Station, TX, USA). The Kruskal-Wallis equality-of-proportions rank test was used to assess differences between facilities and clinician-type, and the number of missing items on referrals. Probabilities were reported with a correction factor for scores with tied ranks. Similarly, the two-sample Wilcoxon rank-sum test was used to determine whether there was a difference between the use of a structured form or no form, and the number of missing items on referrals.

## Results

### Completeness of referrals and use of referral forms

**Figure 1:**
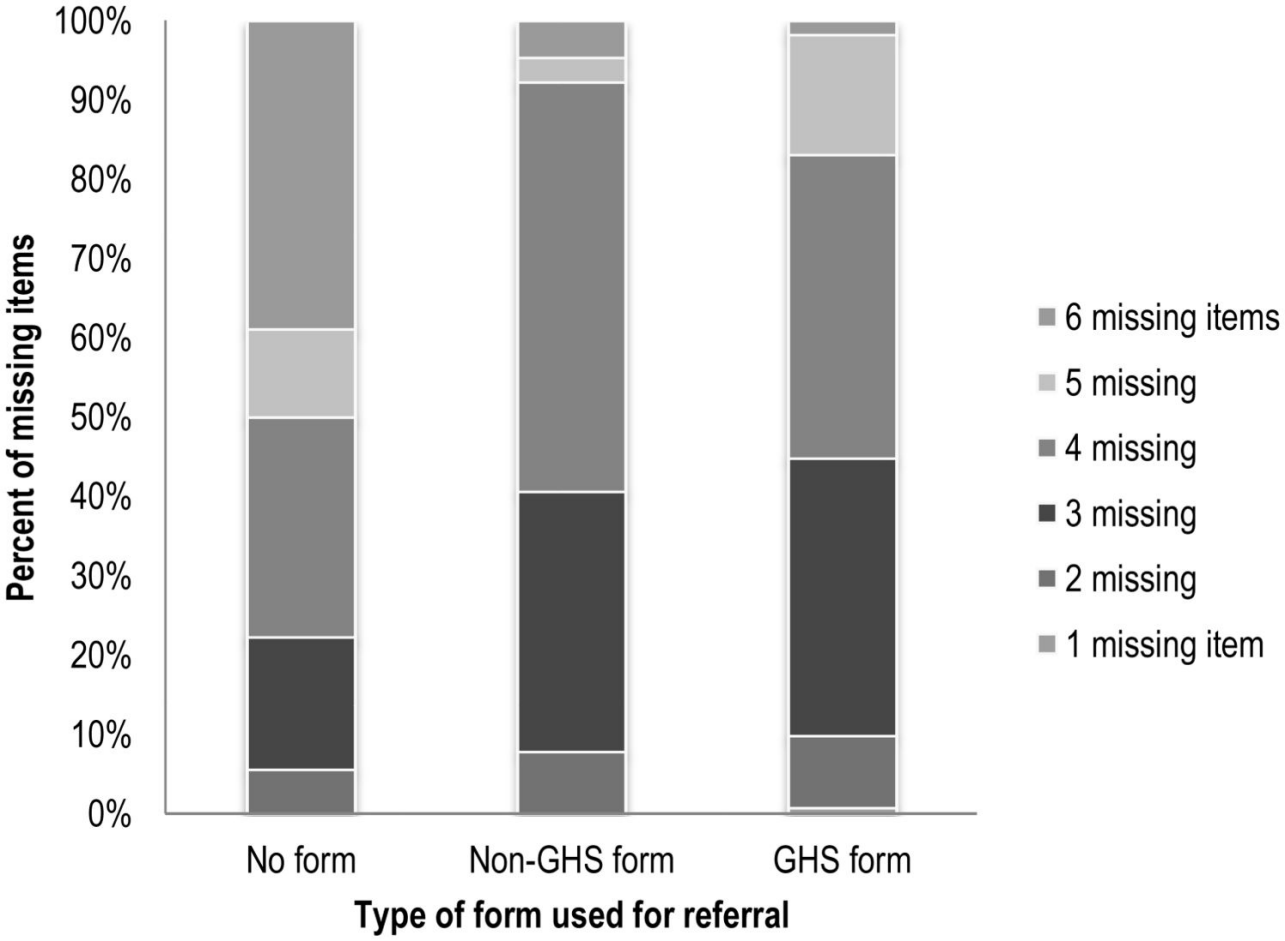
Percent of missing essential items by structured form use on referrals for surgery to Komfo Anokye Teaching Hospital, Ghana.

A total of 643 referrals for surgery were assessed. Of these, none recorded all of the essential information. The median number of missing items was 4 (range 1 – 7). Clinicians who did not use a structured form missed 5 or more essential items 50% of the time, compared with 17% when the GHS form was used and 8% when a non-GHS, but structured form was used. However, even with the use of any structured form, 1 or 2 items were not recorded for 10% of referrals and up to 3 items were not recorded for 45% of referrals (Figure 1). Referrals that used a structured form recorded more items than those that did not use a structured form (*p*=0.001).

### Facility type

Most referrals were either from teaching (45%) or government district hospitals and clinics (26%). Though patient's age, working diagnosis and reason for referral were the most commonly recorded items at all facilities, they too were often missing (5 – 34% of referrals). Though patient's medical history or treatment received for the condition being referred were only recorded for 39 – 58% of referrals, this was markedly more often than the patient's surgical history or respective treatment or diagnostic evaluation prior to referral (2 – 5%) (Table 2). There was no evidence for a difference between facility type and number of missing items (*p*=0.10).

### Clinician type

Most referrals were from physicians (66%); however, physician or medical assistants (PAs or MAs) wrote 14% referrals and providers that did not record their profession (18%) wrote more referrals than nurses or midwives (2%). PAs or MAs (96%) and nurses or midwives (93%) recorded a working diagnosis more often than physicians (75%). The proportion of referrals with recorded surgical history or respective treatment and diagnostic evaluation prior to referral did not vary by clinician type (Table 3). There was weak evidence for a difference between clinician type and number of missing items (*p*=0.06).

## Discussion

This study aimed to assess the quality of referrals for surgery to a tertiary hospital in Ghana, and identify deficiencies in required information essential for timely surgical care. By doing so, effective interventions for improving the surgical referral process could be developed. Referrals uniformly lacked essential information; usually more than 3 of the 7 items. Further, athough the use of a structured form significantly reduced the number of missing items, there remained a substantial proportion of missing items regardless of referring facility or clinician type. In LMICs, the referral form is often the only medical record that accompanies the patient. Thus, provision of timely and appropriate care heavily depends on the quality of information and completeness of the referral form.[33]

The use of structured forms for patient referral from one level of care to the next has been reported to improve communication of essential information in well-resourced healthcare systems.[22, 24, 28, 29] The use of structured referral forms resulted in a 20 – 45% increase in the proportion of non-missing items compared to referrals done without a structured form.[28] Further, referral systems that include standardized forms reduce wait times by as much as 75%, increase patient satisfaction scores, and are associated with improved disease-specific outcomes.[34-36] Given these reports and results from this study, LMIC healthcare systems, which rely heavily on referrals surgical conditions, should consider the use of standardized referral forms to increase communication of information essential for timely, cost- and clinically-effective care.

However, recommending a structured form alone may be inadequate. Even among clinicians who used a structured form, there was a high proportion of missing essential information. To improve compliance with structured form completion, clinicians must understand the patient benefit of referral form compliance.[37, 38] In a study that evaluated colorectal surgery referrals and provided peer-mediated feedback to referrers who did not communicate essential information, clinicians improved their proportion of non-missing items by 25% after feedback.[39]. Further, providing feedback resulting in improved adherence to structured referral forms may reduce inappropriate referrals. Educative feedback to general practitioners not adhering to standard information requested when referring patients in need of gastroscopy was reported to have reduced unnecessary referrals by 31%.[40] Similarly, after structured referral forms became an integrated component of breast care in Cardif, United Kingdom, inappropriate referrals dropped from 55 to 8%.[41] LMIC healthcare systems are already strained with an increasing prevalence of surgical needs.[2, 9] Reducing unnecessary referrals by promoting compliance to structured referral forms that readily allow identification of patients in need of surgery could be another method to improve timely access to surgical care and increase economic efficiency.

Ahough the benefits of healthcare information technology (IT) elude most LMICs, planning IT capacity improvements is becoming a priority.[42] Referral systems built into electronic medical records in HICs ensure compliance with essential items and allow for immediate processing at secondary and tertiary facilities.[22] In Norway, for example, the creation of an electronic surgical referral system reduced waiting times, improved staff efficiency, and required only 37 referred patients to be cost-effective.[43] In healthcare systems with already long waiting times, deficient numbers of staff and limited financial resources, planning electronic referral systems into LMIC IT capacity improvements should be considered. Although IT investment would require significant upfront costs, these would be more than balanced by process efficiencies and cost savings in the long-term.

To our knowledge, this study is the largest examination of referrals from a LMIC and provides important results for improving the referral process. However, there are several limitations. First, there were relatively small numbers of referrals from private or mission hospitals, and nurses or midwives. While there didn't appear to be a difference in missing items between other facilities or clinicians, they may exist and give further guidance to the development of an effective intervention. Second, there are a number of different referral forms in circulation and items that require completion. In an attempt to capture this variation, inclusive assessment criteria were used for a brief history of present illness, medical and surgical history, and diagnostics to date. Further, the item rating criteria allowed for statements that indicated that these have not been performed/asked or the capacity was not available. Lastly, this study did not capture information on the effect of appropriately completed referral forms compared to those with missing items, such as waiting times, cost, or patient satisfaction. Such an examination could provide further evidence for utility of implementing and promoting the use of a structured referral system that may be of great benefit in LMICs where resources for surgical care are concentrated in a very small number of hospitals, necessitating frequent referrals. Despite these limitations, these results allow reasonable conclusions about referral practice in Ghana to be drawn and allow development of targeted interventions to improve surgical referrals locally, and in other LMICs.

## Conclusion

This study identified an opportunity to improve the referral process in Ghana. Structured forms reduced missing essential information on referrals for surgery. However, suggesting that a structured form be used was not enough to ensure consistent communication of essential items. Several potential interventions may improve compliance with appropriately completing structured forms, including: creating a user-friendlier version; implementing a peer-feedback mechanism for poorly completed referral forms; or development of electronic referral systems. Though often not considered among interventions to improve surgical capacity in LMICs, referral process improvements may reduce waiting times, inefficient duplication of scare resources, costs and unsatisfactory results for conditions with time-dependent outcomes.

## Figures and Tables

GHS – Ghana Health Service

**Table 1:** Suggested desired and essential items for surgical referral in low- and middle-income countries.

Desirable	Essential
Date referred	Patient's age
Referrer contact	Working diagnosis
Patient contact	Reason for referral
Gender	Abbreviated history of present illness
Language needed	Diagnostic tests to date
	Medical history or treatment provided
	Surgical history or treatment provided

**Table 2:** Recorded essential items by referring facility type on referrals for surgery to Komfo Anokye Teaching Hospital, Ghana.

	Referring facility type							
	Government		Private		Mission		Teaching	
	n	(%)	n	(%)	n	(%)	n	(%)
**Number of referrals**	167	(26)	86	(13)	97	(15)	291	(45)
**Patient's age**	148	(89)	75	(87)	85	(88)	269	(92)
**Working diagnosis**	148	(89)	71	(83)	82	(85)	193	(66)
**Reason for referral**	139	(84)	73	(85)	85	(88)	277	(95)
**Brief history of illness**	34	(20)	22	(26)	18	(19)	79	(27)
**Medical history or treatment**	96	(58)	36	(42)	55	(57)	112	(39)
**Surgical history or treatment**	5	(3)	4	(5)	2	(2)	12	(4)
**Labs or imaging performed**	5	(3)	4	(5)	4	(4)	6	(2)

An item was considered absent if there was no documentation about the result or its being performed/asked or not performed/asked

**Table 3:** Recorded essential items by referring clinician type on referrals for surgery to Komfo Anokye Teaching Hospital, Ghana.

	Referring clinician type							
	Physician		PA or MA		Not recorded		Nurse or midwife	
	n	(%)	n	(%)	n	(%)	n	(%)
**Number of referrals**	426	(66)	88	(14)	115	(18)	14	(2)
**Patient's age**	388	(91)	12	(86)	104	(90)	12	(86)
**Working diagnosis**	319	(75)	84	(96)	79	(69)	13	(93)
**Reason for referral**	385	(90)	73	(84)	105	(91)	13	(93)
**Brief history of illness**	102	(24)	18	(21)	28	(24)	5	(36)
**Medical history or treatments**	191	(45)	58	(66)	45	(39)	5	(36)
**Surgical history or treatments**	15	(4)	4	(5)	3	(3)	1	(7)
**Labs or imaging performed**	13	(3)	1	(1)	5	(4)	0	(0)

PA – physician assistant; MA – medical assistant; An item was considered absent if there was no documentation about the result or it's being performed/asked or not performed/asked

## Highlights

- Use of structured referral forms improved communication of essential items
- Even with structured forms, essential information was often not conveyed
- Use of structured forms alongside peer-feedback or electronic referral service may improve access to timely care in LMICs
